# Effect of quick simple exercise on non-specific low back pain in Japanese workers: a randomized controlled trial

**DOI:** 10.1265/ehpm.22-00203

**Published:** 2023-06-15

**Authors:** Fuminari Asada, Takuo Nomura, Kenichiro Takano, Masashi Kubota, Motoki Iwasaki, Takayuki Oka, Ko Matsudaira

**Affiliations:** 1 Research Center for the Health Promotion and Employment Support, Osaka Rosai Hospital, Sakai city, Osaka, Japan; 2 Department of Rehabilitation Sciences, Faculty of Allied Health Sciences, Kansai University of Welfare Sciences, 3-11-1 Asahigaoka, Kashiwara city, Osaka, Japan; 3 Research Center for the Health Promotion and Employment Support, Kansai Rosai Hospital, Amagasaki city, Hyogo, Japan; 4 Department of Orthopaedic Surgery, Osaka Rosai Hospital, Sakai city, Osaka, Japan; 5 Department of Medical Research and Management for Musculoskeletal Pain, 22nd Century Medical and Research Center, The University of Tokyo, Tokyo, Japan

**Keywords:** Non-specific low back pain, Quick simple exercise, Occupational health physiotherapy

## Abstract

**Background:**

We designed a quick simple exercise program that can be performed in a short period of time in real-world occupational health settings and investigated the effects of three months of program implementation on non-specific low back pain (NSLBP).

**Methods:**

Participants were 136 individuals working in the manufacturing industry. The quick simple exercise program was designed to be doable in three minutes and consisted of two exercises: a hamstring stretch and a lumbar spine rotation with forward, backward, and lateral flexion. This was a randomized controlled trial incorporating an intervention group to whom the exercises were recommended within a leaflet, and a control group to whom the exercises were not recommended. NSLBP was evaluated at baseline and after three months using numerical rating scale (NRS) scores, ranging from 0 points (no pain at all) to 10 points (worst pain imaginable). The percentages of cases that improved by a minimal clinically important difference (two points or above) were compared.

**Results:**

Overall, 76.1% of the intervention group participants performed the quick simple exercises at least once every one or two days. Three months after baseline, a significantly higher percentage of participants in the intervention group (17 participants: 25%) had NSLBP improvement of two or more points on the NRS compared to that in the control group (8 participants, 12%) (P = 0.047). The average NRS score decreased significantly from 1.87 ± 1.86 to 1.33 ± 1.60 in the intervention group but showed no significant change in the control group, transitioning from 1.46 ± 1.73 to 1.52 ± 1.83. A significant interaction was also observed between the intervention and control groups (F = 6.550, P = 0.012).

**Conclusions:**

Three months of a quick simple exercise program among workers in the manufacturing industry increased the percentage of workers with improvement in the NRS scores. This suggests that the program is effective in managing NSLBP in workers in the manufacturing industry.

**Trial registration:**

UMIN-CTR UMIN000024117.

## Introduction

Low back pain (LBP) is a symptom common to all age groups worldwide, primarily among the working population [1]. The prevalence of LBP among workers aged 18–64 years in the United States is 25.7% [2]. LBP is also a common complaint among the Japanese, with an estimated prevalence of 24.3%–31.7% among those aged 30 years and older [3]. Further, there is likely a fairly constant number of patients with LBP in the workplace considering the combination of existing cases resolving and new cases appearing. Interventions to reduce the rates of LBP complaints must not only improve pain severity, as evaluated using the numeric rating scale (NRS), but also prevent new-onset LBP and stop existing cases from becoming chronic.

Chronic LBP is defined as LBP persisting for at least three months after onset [4, 5]. Chronic LBP increases workers’ fear-avoidance beliefs (FAB) and is associated with presenteeism [6]. For the effective management of chronic LBP, it is important to focus not only on physical issues, but also on psychosocial issues: both psychotherapy and exercise therapy, including full-body exercises and walking, have been shown to be beneficial [7]. However, psychotherapy requires the intervention of trained and specialized medical professionals, while exercise therapy requires time and physical space. Workers in Japan work many hours of overtime and have little free time [8]. Moreover, compared to other countries, occupational health settings in Japan are unlikely to include professionals in musculoskeletal pain, including non-specific LBP [9]. In Japan, LBP countermeasures in the field of occupational health focus primarily on ergonomic interventions for acute LBP, and under the present conditions it would be difficult to establish a system capable of providing psychophysiological interventions and incorporating chronic LBP. There are few professionals capable of distinguishing and properly caring for acute and chronic LBP among workers; therefore, simple countermeasures to address non-specific LBP (NSLBP) are needed.

Thus, there is a need for interventions to prevent NSLBP in occupational health settings. These interventions should not require treatment at a medical institution or special equipment. Accordingly, we assumed that a quick and easy exercise program could become a realistic LBP preventive strategy that is compatible with a busy workstyle. Thus, we developed a quick simple exercise program incorporating stretching and a combined exercise for the lower back. The program can be performed in three minutes and we verified its effects among workers in the manufacturing industry. Sitting or standing for a long time were recognized potential risk factors for LBP in the manufacturing industry [10, 11]. Therefore, we chose workers in the manufacturing industry as our study population. Our previous study found a significant decrease in the NRS score, representing LBP severity [12].

Interventions for LBP typically consist of education provided by a doctor or physical therapist. In previous research, interventions have typically been time-consuming, for example combining information booklets and sessions with a physical therapist for an intervention taking approximately four hours [13] or requiring daily twenty-minute yoga or exercise classes [14]. For the intervention in the present study, we distributed leaflets recommending three minutes of exercise, allowing the intervention to be completed in a short period of time similar to that of the “One Stretch” exercise [15, 16]. In this study, we distributed leaflets recommending quick simple exercises that we developed for workers in the manufacturing industry and evaluated items related to LBP after three months. The goal was to determine whether the quick simple exercise program was effective in managing the rate of LBP complaints among workers in the manufacturing industry.

## Methods

### Participants and study design

This study was conducted as a preventive care model research project of the Japan Labor Health and Welfare Organization with the goal of developing measures to prevent LBP in the workplace. The sample constituted 128 individuals (64 per group) based on the sample size obtained using G*power version 3.1.9.7 (Heinrich-Heine-Universität Düsseldorf, Germany) [17]. Based on the LBP values derived from our previous study [13], an effect size of 0.25 was calculated to achieve an α error probability of 0.05, a power (1−β error probability) of 0.8, and a number of covariates of 1 within the family of F tests; statistical test of the repeated measures analysis of covariance (ANCOVA) was performed.

This was a randomized controlled trial. A short class on preventing LBP was taught by a physical therapist in December 2016 and July 2018. Participants provided written consent to participate after a summary of the research was explained at Companies A (Izumisano city, Osaka prefecture) and B (Amagasaki city, Hyogo prefecture). The inclusion criteria were as follows: age 20–65 years and participation in the short course regardless of the presence of LBP. The exclusion criteria were as follows: diagnosis of specific LBP and ongoing treatment, ongoing treatment for mental illness or other decline in mental health, presence of any illness that limited movement, lack of consent for participation in the study, and pregnancy. The criteria for discontinuation of study participation were as follows: inability to continue exercising due to severe illness complications, difficulty with study participation due to mental health reasons, transfer to a workplace in a different region or retiring, becoming pregnant, withdrawal of consent, and missing questionnaire responses.

**Fig. 1:**
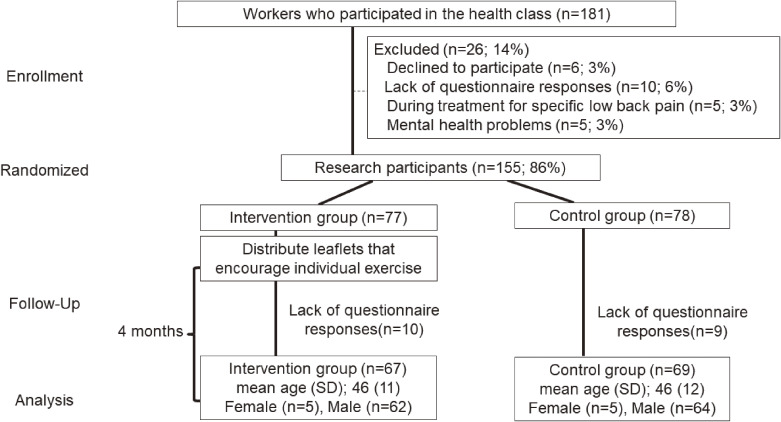
Participant flow in this study SD, standard deviation

The intervention was performed in a three-month period between December 2016 and April 2017 at Company A and between July 2018 and November 2018 at Company B. Quick and simple whole-body exercises were recommended before work in each workplace. However, these exercises were not aimed at preventing LBP. Their purpose was unclear, and practicing them was not compulsory. There were 154 and 27 participants in the physical therapist’s short courses at Companies A and B, respectively. Questionnaires were distributed to these 181 individuals, and a total of 165 responses were collected after the exclusion of non-responses and incomplete responses (response rate, 92%). Questionnaire data for those with a history of diagnosis and treatment for specific LBP (5 participants) or poor mental health (5 participants) were removed. The data for the remaining 155 participants were randomly assigned to two groups after adjusting for age and sex. Assignment was performed using a computer-generated random number table by an individual not involved in the evaluation or instruction. Participants as well as researchers responsible for measurement and analysis were blinded to participant assignment. Leaflets were distributed to participants individually in envelopes with their names. A total of 77 participants were assigned to the intervention group (mean age 46 ± 12 years, 71 men, 5 women) and 78 to the control group (mean age, 46 ± 12 years; 70 men and 8 women; BMI, 23.3 ± 3.1 kg/m^2^). Baseline and three-month questionnaires were numbered in advance with numbers linked to the assignment table, and questionnaire data were input by administrative staff at the researchers’ institutions. Input data were coded with 1 and 2 representing the intervention and control groups, respectively, to blind the researchers performing analysis (Fig. 1).

This study was approved by the ethics committee of Osaka Rosai Hospital (approval no. 27-115) and registered in the UMIN Clinical Trials Registry (UMIN000024117).

### Outcome measures

The primary outcome was LBP experienced over the past four weeks as measured on the NRS, ranging from 0 (no pain at all) to 10 points (worst pain imaginable) [18]. The percentages of participants with NRS score improvement of a minimal clinically important difference (MCID) [19, 20] (decrease by at least two points [21, 22]), no change (±1 point), or worsening were compared. The secondary outcome was health-related quality of life, which was measured using EuroQol 5 Dimensions (EQ-5D) and its utility value calculated [23]. These were measured at baseline and after three months.

The following items were measured at baseline to compare baseline characteristics and job status between the intervention and control groups. For general characteristics, sex, age, height, weight, and smoking status were collected. For LBP-related medical history and psychosocial information, history of medical examination for LBP, presence of LBP in the past year, depression/anxiety disorder, and FAB were collected. Depression/anxiety disorders were classified based on a score of ≤4 points (no mental health problems) or ≥5 points (mental health problems) on the K6 [24]. Fear-avoidance beliefs were classified based on a score of ≤14 points (no problems) and ≥15 points (severe FAB) on the Japanese version of the FAB Questionnaire (FABQ) physical activity scale [25]. For job status, data on whether the participant held an administrative position, was a full-time staff member, worked night shifts, their type of work, work performance over the past 30 days, and hours worked per week, including overtime, over the past month were collected. Presenteeism was measured using the Japanese version of the World Health Organization’s Health and Work Performance Questionnaire [26, 27].

### Quick simple exercises

**Fig. 2:**
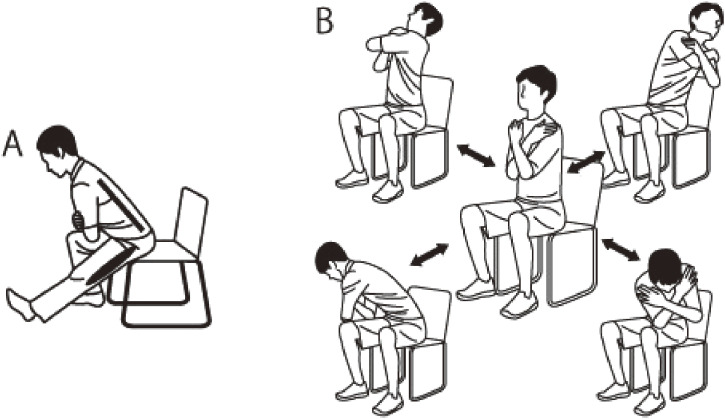
Details of quick simple exercise A. Hamstring stretch B. Lumbar spine rotation with forward, backward, and lateral flexion

The quick simple exercise program was designed to be performed within three minutes and comprised two exercises—a hamstring stretch and a lumbar spine rotation with forward, backward, and lateral flexion (Fig. 2).

Hamstring stretch: While sitting on the edge of a chair, extend one leg out in front and incline the upper body forward as far as possible without pain, taking care not to bend the upper body too far forward. Hold this position for twenty seconds and perform three sets per leg.

Exercise with anterior-posterior and lateral flexion in addition to trunk rotation: Sit shallowly on a chair with both lower limbs shoulder-width apart and both upper limbs crossed in front of the chest. The following four movements in four directions are performed once: 1) right trunk rotation, right lateral flexion, and back bending (extension); 2) left trunk rotation, left lateral flexion, and back bending (extension); 3) right trunk rotation, right lateral flexion, and forward bending (flexion); and 4) left trunk rotation, left lateral flexion, and forward bending (flexion). When performing the movements (1) to (4), check to see if pain appears during any of the four movements, while avoiding forceful movement in the direction that causes pain. Next, movement in all directions, except those in which no pain appears, should be performed. Perform the exercise 5 to 10 times in one direction at a comfortable speed. Then, move the trunk in the direction in which the pain appeared earlier, and self-evaluate the degree of pain, the angle at which it appears, and its location.

### Procedure

The workshops for LBP prevention consisted of a one-hour lecture by a physical therapist on the causes of LBP; the relationship between LBP and ergonomic factors, such as posture and movement; the relationship between LBP and psychosocial factors; methods of self-assessment for LBP risk; and important parts of LBP prevention. Baseline data were collected using a questionnaire from a total of 136 participants from Companies A and B, who provided written and oral informed consent to participate in the study. Research participants were informed that they would be classified into either an intervention or a control group. The participants later underwent stratified assignment based on sex, age, and presence of NSLBP in the past year, with both companies comprising intervention and control groups. Both groups were mailed a pamphlet with a written summary of the content of the workshop, and the pamphlet for the intervention group also included details of the quick simple exercise program and instructed participants to perform the quick simple exercises at least once every day at work.

After three months, the same data that were collected at baseline were collected again with the assistance of the health and safety supervisor at each company. In the post-intervention evaluation, participants in the intervention group were also asked how often they performed the physical exercises during a five-day work week using the following question: “Did you do the quick simple exercises every day?” Response options were as follows: (1) “I did them on at least four days,” (2) “I did them on about three or four days,” (3) “I did them on two or three days (about once every two days),” (4) “I did them on about one or two days (about once every three days),” (5) “I did them on about one day,” and (6) “I did not do them at all.”

### Statistical analysis

Differences between the intervention and control groups for continuous variables were evaluated based on the results of the Shapiro–Wilk test using the t-test or Mann–Whitney U test. Nominal variables used the chi-squared test or Z-test. Two points was considered a clinically important change on the NRS. First, the percentages of participants with an NRS score of at least two points at baseline were compared. Then, the percentages of participants with improvements of at least two points on the NRS were compared from baseline to three months later between the intervention and control groups using the chi-squared test.

Using the Wilcoxon signed-rank test, changes in NSLBP over the past four weeks, as measured by the NRS score and EQ-5D utility value, were compared from baseline to three months later separately in the intervention and control groups. Next, the analysis of covariance (ANCOVA) was performed to investigate NRS interactions from baseline to three months later in the intervention and control groups. The covariate for ANCOVA was the presence or absence of LBP over the past year.

Statistical analyses were performed using IBM SPSS Statistics, ver. 24 (IBM Corp., Armonk, N.Y., USA), with a significance level of 5%.

## Results

There were no significant differences in the general characteristics, history of NSLBP, psychosocial information, or job status between the intervention and control groups (Table 1). Three months after baseline, a significantly higher percentage of participants in the intervention group (17 participants, 25%) had improvements of two or more points on the NRS compared to that in the control group (8 participants, 12%) (P = 0.047).

**Table 1:** Characteristics, low back pain related parameters, and job status of participants at baseline

**Parameters**	**Units**	**Intervention group**	**Control group**	***P*-value**
Sex (male/female)	n	62/5	64/5	0.609
Age	years	46.1 ± 11.1	45.7 ± 12.1	0.939
Body height	cm	170.4 ± 6.5	170.2 ± 7.5	0.877
Body weight	kg	68.3 ± 11.0	66.0 ± 9.4	0.271
Body mass index	kg/m^2^	23.4 ± 3.0	22.7 ± 2.8	0.170
Current smoker	*n* (%)	22 (32.8)	20 (29.0)	0.711
Past medical history of NSLBP	*n* (%)	12 (17.9)	8 (11.6)	0.340
Presence of NSLBP before 1 year	*n* (%)	50 (74.6)	46 (66.7)	0.350
NRS of 2 or more	*n* (%)	37 (57.8)	27 (42.2)	0.085
Depression/anxiety disorder	*n* (%)	5 (7.5)	6 (8.7)	1.000
15 points over of FABQ	*n* (%)	8 (11.9)	15 (21.7)	0.170
Job status				
Administrative position	*n* (%)	10 (15.2)	18 (26.1)	0.140
Full time staff	*n* (%)	59 (88.1)	59 (85.5)	0.801
To get nighttime shift	*n* (%)	17 (25.4)	22 (31.9)	0.451
Desk worker	*n* (%)	27 (40.3)	28 (40.6)	1.000
Single-item presenteeism question*	points	6.1 ± 2.0	6.2 ± 1.8	0.609
Average working time				0.419
Under 40 hours	*n* (%)	10 (14.9)	16 (23.2)	
40 to 50 hours	*n* (%)	37 (55.2)	38 (55.1)	
50 to 60 hours	*n* (%)	19 (28.4)	13 (18.8)	
60 hours and over	*n* (%)	1 (1.5)	2 (2.9)	

Data are presented as mean ± SD or n (%). NSLBP, non-specific low back pain; NRS, numerical rating scale; FABQ, fear-avoidance beliefs questionnaire. Single-item presenteeism question*: To evaluate presenteeism, we modified the World Health Organization Health and Occupational Performance Questionnaire for the Japanese participants.

Overall, 76.1% of the intervention group participants performed the quick simple exercises about once every two or one days over the three-month period. The details of exercise practice frequency are as follows: more than four days, n = 23 (34.3%); two or three days, n = 23 (34.3%); one or two days, n = 5 (7.5%); one day, n = 5 (7.5%); did not do, n = 11 (16.4%). Table 2 shows changes from baseline to three months later for the intervention and control groups. First, there was no significant difference in NSLBP NRS score or EQ-5D utility value between the intervention and control groups at baseline. Concerning changes from baseline to three months later in the control group, the NRS score transitioned from 1.46 ± 1.7 to 1.52 ± 1.8 and the utility value from 0.89 ± 0.13 to 0.90 ± 0.13, showing no significant differences. Concerning changes from baseline to three months later in the intervention group, the utility value transitioned from 0.86 ± 0.14 to 0.89 ± 0.13, showing no significant difference. However, the NRS score in the intervention group significantly improved from 1.87 ± 1.86 at baseline to 1.33 ± 1.60 three months later (P = 0.002) and showed a significant interaction with the control group (F = 6.550, P = 0.012).

**Table 2:** Changes in non-specific low back pain and health related quality of life after 3 months

**Parameters**	**Units**	**Group**	**Baseline**	**After 3 months**	**Interaction**	

					***F*-value**	***P*-value**
NSLBP intensity	point	Intervention group	1.87 ± 1.86	1.33 ± 1.60^†^	6.550	0.012
		Control group	1.46 ± 1.73	1.52 ± 1.83		
EQ-5D	scores	Intervention group	0.86 ± 0.14	0.89 ± 0.13	0.481	0.489
		Control group	0.89 ± 0.13	0.90 ± 0.13		

Data are presented as mean ± SD. NSLBP, non-specific low back pain. Parameters of the repeated measures analysis of covariance (ANCOVA): the covariate was the presence of NSLBP before 1 year.

†P < 0.002 in the intervention or control group.

## Discussion

This study investigated the effect of the distribution of leaflets recommending a quick simple exercise program developed by the authors on reducing LBP three months later among workers in the manufacturing industry. Our results demonstrated a significant improvement in LBP NRS scores in the intervention group and a significant interaction with the control group. As not all subjects in the present study had LBP, NRS score changes were compared with consideration to the MCID. Consequently, the percentage of participants who showed improvement was significantly higher in the intervention group than in the control group.

The prevalence of LBP complaints in Japan ranges from roughly 10%–15% to 35%–39% [28, 29], and surveys by occupation report rates of 30% for nurses, 22% for desk workers, 19% for workers in sales, and 31% for the transportation industry [30]. This suggests that workers with more physically strenuous jobs are more likely to complain of LBP. Roughly 20% of factory employees who work in the standing position complain of LBP [31]. As the present study focused on workers in the manufacturing industry who participated in a workshop on strategies for preventing LBP, many participants struggled with LBP on a daily basis or had an interest in preventing LBP among the employees they manage. As a result, the rate of LBP complaints at baseline was 64% in the intervention group and 59% in the control group.

Return to the workplace is faster with NSLBP than with organic LBP [32], and factors delaying workplace reinstatement include radiating pain, severity of functional disability, high work tempo and work quantity, and interpersonal relationships with coworkers [33]. A study investigating the effect of a placebo drug on NRS score changes among Japanese patients with chronic pain found no significant effect compared to the control group [34]. A study in which Japanese subjects were assigned to either simple and brief or materials-based 100-minute education found no additional improvement in the NRS score for the simple and brief education but did confirm additional improvement in functional limitation, self-efficacy, and EQ-5D [35]. In contrast, there was no significant difference in health-related QOL before and after the intervention in our study. The QALY was comparatively high in the intervention group (0.86 ± 0.14 at baseline) and the control group (0.89 ± 0.13 at baseline). We considered this a ceiling effect, not that the exercises were ineffective.

In the present study, 76.1% of the intervention group participants performed the quick, simple exercises about once every one or two days over the three months. Before our intervention, practicing quick and simple whole-body exercises before work had been recommended at the workplaces. However, these exercises were not aimed at preventing LBP; their purpose was unclear. Also, the exercises were not compulsory. Zunft et al. reported that the most important motivation for people to engage in physical activity is to maintain good health, release tension, and get fit [36]. In the present study, we clarified the purpose of the exercises to the intervention group. Quick, simple exercises may have resulted in real-time low back relaxation. Furthermore, smart physiological feedback may have been provided by practicing the exercise itself. These factors may have raised the practice frequency of exercises. However, it was unclear whether a high practice frequency of simple, quick exercise led to great LBP improvement.

Exercise therapy for chronic LBP has been shown to improve pain levels to the same extent as anti-inflammatory drugs and to be more effective for functional disability [37]. Exercise is also essential for supplying nutrients to the intervertebral discs of the lumbar spine [38]. A review utilizing the Cochrane database found that exercise therapy to treat NSLBP improved the pain level by 7.3 points out of 100 (95% CI, 3.7 to 10.9) regardless of whether the pain was acute, sub-acute, or chronic [39]. Analysis in the present study also included individuals without LBP, but the intervention group score of 5.4 points suggests that the intervention resulted in improvement, even if the score was less. Psychological improvement (e.g., FABQ) is important for interventions for chronic LBP, but the intervention through leaflet distribution implemented in the present study did not impact psychological evaluations or presenteeism.

The present study had some limitations. First, the study did not provide evidence of the mechanism by which the exercise program developed by the authors improves LBP, and thus discussion of the reduction of LBP is only speculative. Second, because the participants in the present study had participated in a workshop on LBP prevention, they already had some knowledge of LBP prevention before performing the quick simple exercises. Having systematic knowledge of steps to prevent LBP may have influenced catastrophic thinking about pain and contributed to LBP reduction. However, we were unable to verify this in detail, and our study showed no change in FABQ. Third, continuous poor posture, such as a forward bending of the trunk (lumbar kyphosis), makes LBP more likely, while exercises that require backward bending of the trunk (lumbar lordosis) may prevent or improve LBP. The exercises performed in the present study included backward bending of the trunk, but these exercises were not compared with others. In the future, it is necessary to examine the occupations in which exercise effects are observed and the persistence of the effects; further, it is also necessary to verify the differences between exercises for which effects are observed and confirm the synergistic effects of exercises. Finally, future studies need to examine the influence of different working environments such as the time spent standing and the working posture, health-related QOL, and practice frequency of quick, simple exercises on NSLBP improvement.

## Conclusion

The prevention and treatment of LBP require integrated health and occupational interventions that reduce physical strain at work, as well as public health and prevention strategies [40]. Exercise, by itself or in combination with education, is reported to reduce the risk of LBP [41, 42]. In occupational LBP prevention as well, exercise decreases pain severity and functional disability [43]. The simple program we developed will be helpful in preventing NSLBP in occupational health settings. Lastly, it is essential that strategies for preventing chronic LBP and other forms of LBP simultaneously promote strategies for addressing psychosocial factors. Thus, it is necessary to consider the effects on psychosocial factors in addition to exercise going forward.

## Abbreviations

ANCOVA: analysis of covariance
FAB: fear-avoidance beliefs
FABQ: fear-avoidance beliefs questionnaire
LBP: low back pain
MCID: minimal clinically important difference
NRS: numerical rating scale
NSLBP: non-specific low back pain

## Declarations

### Availability of data and materials

The datasets supporting the conclusions of this article are available from the corresponding author upon reasonable request.
